# Highly Efficient Clinical Trials Simulator (HECT): Software application for planning and simulating platform adaptive trials

**DOI:** 10.12688/gatesopenres.12912.2

**Published:** 2019-03-18

**Authors:** Kristian Thorlund, Shirin Golchi, Jonas Haggstrom, Edward Mills

**Affiliations:** 1 MTEK Sciences Inc., Vancouver, British Columbia (BC), V5Z 1J5, Canada

**Keywords:** Platform trial, adaptive design, trial simulation, highly efficient clinical trials, open-source software.

## Abstract

**Background:** Adaptive designs and platform designs are among two common clinical trial innovations that are increasingly being used to manage medical intervention portfolios and attain faster regulatory approvals. Planning of adaptive and platform trials necessitate simulations to understand how a set of adaptation rules will likely affect the properties of the trial. Clinical trial simulations, however, remain a black box to many clinical trials researchers who are not statisticians.

**Results: **In this article we introduce a simple intuitive open-source browser-based clinical trial simulator for planning adaptive and platform trials. The software application is implemented in *RShiny *and features a graphical user interface that allows the user to set key clinical trial parameters and explore multiple scenarios such as varying treatment effects, control response and adherence, as well as number of interim looks and adaptation rules. The software provides simulation options for a number of designs such as dropping treatment arms for futility, adding a new treatment arm (i.e., platform design), and stopping a trial early based on superiority. All available adaptations are based on underlying Bayesian probabilities. The software comes with a number of graphical outputs to examine properties of individual simulated trials. The main output is a comparison of trial design performance across several simulations, graphically summarizing type I error (false positive risk), power, and expected cost/time to completion of the considered designs.

**Conclusion: **We have developed and validated an intuitive highly efficient clinical trial simulator for planning of clinical trials. The software is open-source and caters to clinical trial investigators who do not have the statistical capacity for trial simulations available in their team. The software can be accessed via any web browser via the following link: *https://mtek.shinyapps.io/hect/*

## Introduction

Over the past two decades randomized clinical trials have become increasingly innovative ^1^. The surge in innovative designs stems from an increasing need to reduce waste and improve efficiencies in time and cost. Adaptive designs and platform designs are among two common clinical trial innovations that are increasingly being used by the pharmaceutical industry to manage their drug portfolios and get to faster regulatory approvals of new treatments ^2– 4^. These types of trials, when designed appropriately, also have ethical advantages such as reducing the number of patients exposed to an inferior or harmful treatment.

Contrary to conventional randomized clinical trials where all patients are followed-up after a fixed period of time and the pre-planned trial protocol is adhered without deviation, adaptive trials and platform designs allow for pre-planned (and occasionally post-initiation) modifications to the protocol in the event of strong early treatment response signals ^4^. At face value, this makes the properties of adaptive and platform trials difficult to understand because trial investigators do not know up front whether or which pre-planned adaptations may take place over the course of a trial. Hitherto, planning of adaptive and platform trials necessitate simulations to understand how a set of adaptation rules will likely effect the properties of the trial (e.g., the probability of the detecting a true effect) ^5^.

Simulations, whether for clinical trials or other disciplines, are a complex branch of statistics and probability theory, and thus naturally, remain a black box to most clinical trial investigators. In the pharmaceutical industry, expensive comprehensive software packages (e.g., *FACTS ^TM^* or *ADD-PLAN ^®^*) ^6, 7^ are often used to run simulations, but the technical level required to use such packages is typically that of a master’s degree in statistics (or similar) with years of experience. In other areas such as academia where funding typically is not available for these software packages, statisticians will either need to hard code simulations from scratch or code via available packages in statistical software (e.g., the *ADCT: Adaptive Design in Clinical Trials package* for *R*) ^8^. Arguably, both options contribute further to the black box perception and thus are not helpful in the education and promotion of innovative clinical trial designs.

In global health, the Bill & Melinda Gates Foundation is pushing for the use of Highly Efficient Clinical Trials (HECT) – trials where investigators are open to adaptation, where trial simulation is a natural part of the planning, and where the scope of the trial can be altered to sustain local infrastructure and leave a foot print ^9^. Platform adaptive trial designs fit well into this context, but the use of these designs may be hampered by limited access to methodologists with capabilities in trial simulations. To address these limitations, the *Knowledge Integration (KI) trial services* division at The Bill & Melinda Gates Foundation initiated the development of an open source software application with a simple user interface to complete platform and adaptive trial simulations. The software was developed between May 2017 and October 2018 and caters to clinical trial investigators, clinical trial methodologist, and researchers who are not statisticians or do not have access to commercial trial simulator software. This article documents the implementation, methods and functions of the software application.

## Implementation

The Highly Efficient Clinical Trials (HECT) simulator is a web application written in *RShiny*, a package in the statistical software *R* and *RStudio* ^10, 11^. The HECT simulator is compiled to run on any browser. The HECT simulator requires a few manual inputs in the input bar (see also next section) to run. The software allows for simulation outputs to be saved and loaded. The simulation output can be saved in a temporary folder that accompanies the application for online exploration and comparisons. This folder is cleared on a daily basis to prevent accumulation of data. To save the simulation output permanently the user needs to use the download button which downloads the results in form of a table with rows and columns specified by the user.

The clinical trial adaptation rules implemented in the software are based on the calculation of Bayesian posterior probabilities of superiority and inferiority ^12^. These are hardcoded in *R* and further technical details can be found in the software manual’s appendix.

### Overview of inputs and functions

**Figure 1.**
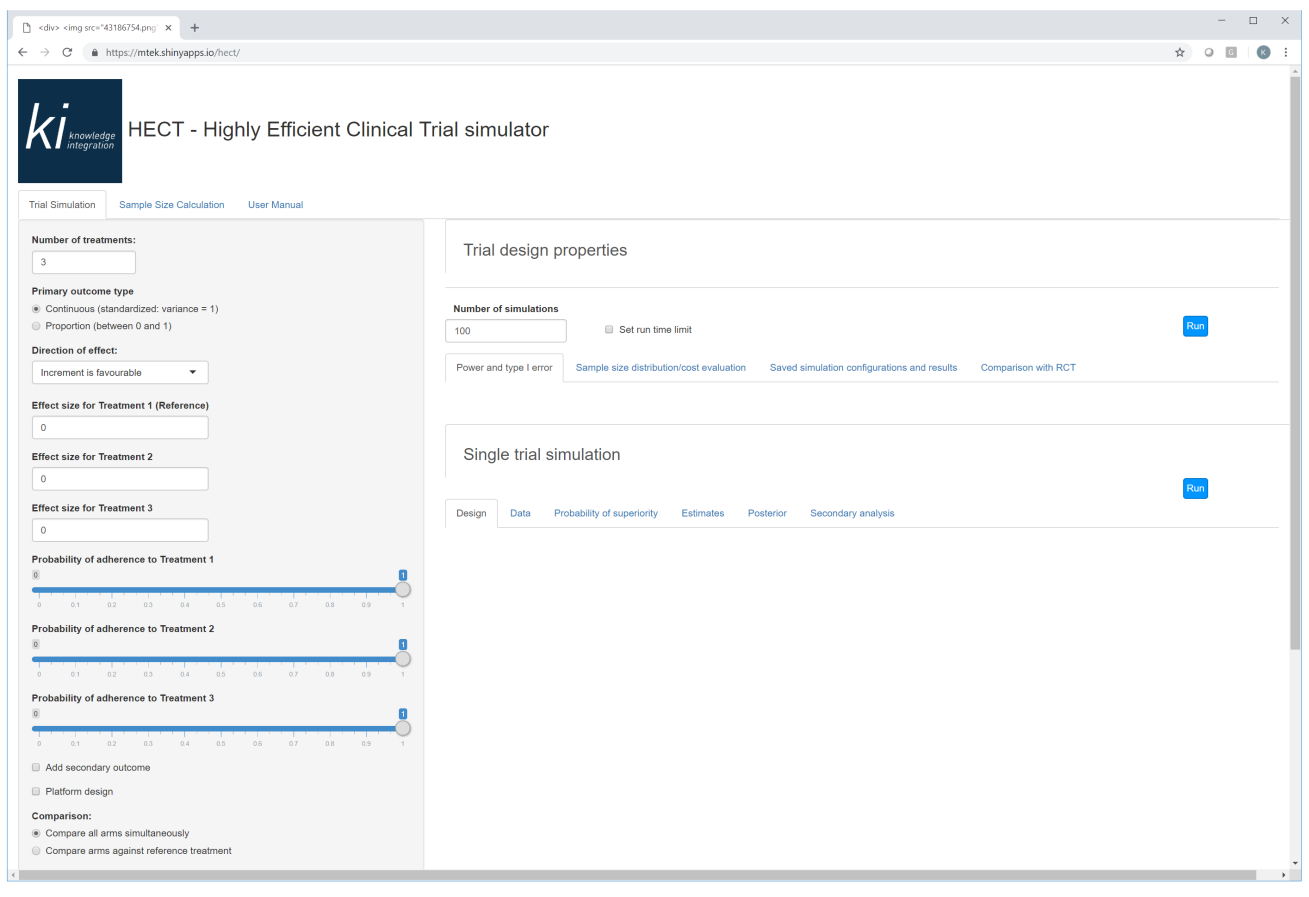
Displays the start up window of the HECT simulator software application

The HECT software application comes with a set of functions and outputs for trial simulation and conventional sample size calculations. The software also provides a brief user manual. Figure 1 shows the opening window of the HECT software. The trial simulator inputs and outputs is found in the first tab to the left, the conventional sample size calculator is available in the middle tab, and the user manual is found in the third tab to the right. The manual outlines each of the input options individually and provides summaries of available outputs. The manual appendix includes a detailed account of the statistical methods used at the back-end of the software. For all functions, the input bar is found to the left in the browser window, and the outputs are obtained and found in the right side of the browser window.

### Clinical trial designs implemented

**Figure 2.**
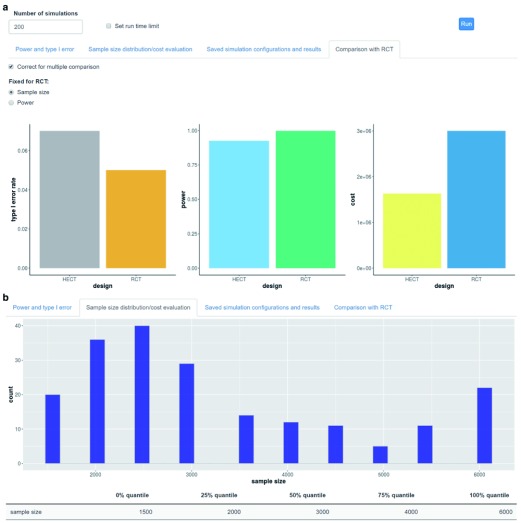
Displays example trial design performance outputs after 200 simulated trials: ( **a**) comparing the type 1 error, power and overall cost between the highly efficient clinical trial (HECT) and the conventional clinical trial design; and ( **b**) the distribution of sample size at trial termination with the highly efficient design (compared to 6,000 for all conventional designs).

***Conventional trial design.*** For comparative purposes, all simulations will automatically incorporate simulations of a conventional 1:1 randomized clinical trial. The conventional trial will accumulate the maximum allowed number of patients in the simulation, which can be informed by a conventional clinical trial sample size calculation (see *Conventional sample size calculation* under *Statistical methods implementation*). Figure 2a display an example of a standard multiple simulations output figure from the software where key properties such as type I error, power, and cost, are compared between a simulated HECT and a conventional trial.

***Early stopping of trial for efficacy.*** The software includes an option to stop the trial early if a strong signal of superiority of one treatment over the other(s) is observed at an interim analysis. The signal is determined by a pre-set threshold for a Bayesian posterior probability of superiority. For example, if the threshold is set to 99%, a single simulated trial will be terminated at the first interim analysis where the probability of any treatment being superior to all other treatment exceeds 99%.

***Early stopping or dropping of treatment arm for futility.*** The HECT software comes with two options for dropping a treatment arm for futility. For two-arm trials, early confirmation of futility may also lead to termination of the trial. First, a treatment arm may be dropped early for futility if the probability that it is superior to all other treatments fall below some pre-set threshold. For example, if the threshold is set to 1%, the treatment arm will be dropped at the first interim analysis where the probability of it being superior to all other treatments falls below 1%. Second, a treatment arm may be dropped with respect to some margin of observed treatment effect, typically an agreed upon minimally clinically important effect. For example, if we consider a relative risk reduction of 20% the minimally important effect for some clinical outcome, we can drop a treatment arm if we are highly certain the treatment effect (compared to control) does not exceed 20%. For example, investigators can insist on being 95% certain the effect is less than minimally important. In this case, the treatment arm will be dropped at the first interim analysis when there is a 95% probability or greater that the relative risk reduction is smaller than 20%.

***Platform trial – adding new treatment arm.*** Similar to dropping a treatment arm, the HECT simulator also allows for the addition of a new treatment arm. This corresponds to using a platform design. The software allows the addition of a new arm to be triggered in two ways. First, if another treatment arm is dropped for futility (see above subsection) a new treatment arm will replace the dropped one. Second, if all arms are dropped except for one, a new arm can be added to compare to the winner of the previous stage. For the latter, this occurs if a trial is stopped for superiority of one treatment, except the platform trial design option allows for a perpetual continuation of the trial into a two-arm comparison.

### Statistical methods implemented

***Conventional sample size calculation.*** As a basis for comparison, the HECT simulator includes a sample size calculator for conventional randomized clinical trials (see appendix of User Manual). For multi-arm trials, the sample size is specified to detect the difference between the largest and second largest effect. The sample size calculator also allows for adjustment of multiplicity for multi-arm trials. The sample size calculator is found in the middle tab.

***Adaptive design stopping rules.*** All stopping rules, whether for treatment arms or the whole trial, are based on the calculation of Bayesian posterior probabilities. Currently (Nov 2018), the software facilitates trial simulation for binary and continuous outcomes. As such, the Bayesian models comprise a conjugate Gaussian model for continuous outcomes and a beta-binomial model (i.e. a beta prior and a binomial likelihood) for binary outcomes. In both models a diffuse non-informative prior is specified.

***Response adaptive allocation.*** Response adaptive adaptation is an optional feature under all trial designs implemented in the HECT simulator. With response adaptive allocation the allocation ratio between treatments is adapted based on which treatment appears most likely to be superior. In other words, on average more patients will be allocated to the treatment with the highest Bayesian probability of superiority ^12^. This method is particularly used when there is a strong incentive to reduce the number of patients exposed to an inferior treatment. While there are many methods of adjusting the allocation ratio, the HECT simulator uses the ratio between square roots of the posterior probabilities of being superior for each treatment ^13^. When response adaptive allocation is selected, the allocation rate is set to equal for all arms (e.g., 1:1 for 2-arm trial) up till a pre-specified ‘burn-in’ sample size and is subsequently altered for every accumulated patient.

### Simulation analysis functions implemented

The HECT trial simulator allows the user to estimate the trial design properties via simulation of large number of trials as well as simulating individual clinical trials to gain a better understanding of within trial variability.

***Trial design properties (multiple trials simulation).*** To evaluate the overall trial design properties such as type I error, power, expected time to trial termination/average sample size, expected costs, etc. it is necessary to simulate multiple trials and average the performance metrics of these. The trial design properties function does just that. Figure 2 illustrates an example of the graphical outputs provided under the Trial Design Properties function. The user can decide how many trials to simulate or for how long the simulation can run (time maximum defined by number of seconds). The user can inspect the overall (or treatment vs control specific) power and type I error (reported numbers), the distribution of final sample sizes and cost across simulated trials (histogram display), as well as the comparison of power, type I error and expected costs between the HECT trial and a conventional design trial (bar plot display). Lastly, the user can save all or some of the recorded variables for the simulation that has just been run.

**Figure 3.**
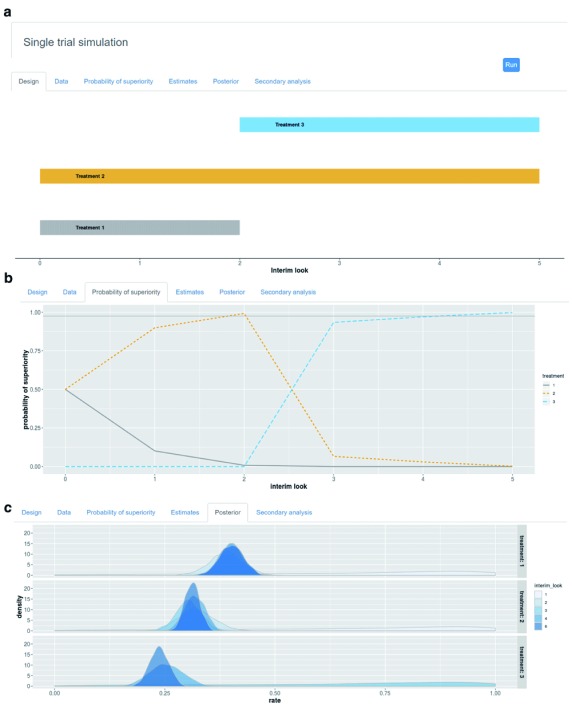
Displays example single trial outputs for: ( **a**) actualized (platform) trial design scheme; ( **b**) the probabilities of superiority for each treatment by interim look; and ( **c**) the probability densities for each treatment by interim look.

***Single trial simulation.*** The single trial simulation function provides several graphical and numerical outputs to inspect within trial trends and variability. Figure 3 illustrates an example of some of graphical outputs provided for single trial simulation. First, the single trial simulation function produces a diagram for the trial flow over time (accumulation of patients) which allows visual representation of when any treatment was dropped or added. Second, a visual representation of where data points fall over time is available (extension of the trial flow diagram). Third, the single trial simulator provides visual inspection of the probabilities of superiority for each treatment over time, specifically at each planned interim look. This option is also available as graphical representation of posterior distributions for each treatment arm at selected interim looks. Finally, the final treatment estimates both for the primary outcome (which adaptations are based on) and optionally some secondary outcome (only monitored) are available to inspect.

### User interface

The user interface comprises of three tabs: Trial Simulation, Sample Size Calculation, User Manual. The latter is a brief account of all input options and general outputs. In addition to the user manual, individual explanations are available for several functions when the user hovers the mouse cursor over the function. Both the Trial Simulation and Sample Size Calculation tabs have their input field to the left and the output to the right. At start up, default values are set to avoid empty values. Quality checks are automatically run, and error messages with accompanying instructions will be provided if the user attempts a computation based on input values that are either beyond the allowed range or of the wrong format.

Where radio buttons are used to define the input format (binary or continuous outcomes) for trial design type (compare all arms or compare vs control), the input field will automatically change to match the selected.

The right side panels are for the outputs. For the Sample Size Calculations tab, the ‘Calculate’ button will initiate the conventional sample size calculation and if all inputs are of correct format, the resulting sample size will appear. For the Trial Simulation tab, the right side is split in two panels. The lower panel is for running single trial simulations (one at a time) and inspecting the within trial behaviour. The upper panel is for summarizing the performance properties across multiple trial simulations. In both, the user is required to press the ‘Run’ button for a simulation (single or multiple) to run. A single trial simulation typically only requires a few seconds, whereas multiple trial simulations may require a few minutes. A progress bar will appear in the lower right corner so the user can follow the back-end computations progress. Whether single or multiple simulations are run, the available output graphs will update once the computations are complete.

## Operation

The HECT simulator is compiled to run on any browser and can be accessed via any web browser using the following link: *https://mtek.shinyapps.io/hect/*. All computations are conducted remotely on an *RShiny* server. The graphical layout of the software is automatically determined by the size of the browser window and the screen resolution. For example, for a 13-inch laptop manufactured in 2018 we recommend you maximize your browser window and use a high resolution setting the first time you open the software.

## Validation and use cases

The functions of HECT simulator software has gone through multiple rounds of validation. At the time of writing (January 14, 2019), the software (including earlier versions of the raw statistical methods code and user interface) has been used in conjunction with early stage portfolio planning. To this end, the software has been used to examine the likely costs and probabilities of success for a large number of candidate designs under various scenarios for possible target countries. An earlier version of the software was beta-tested and independently used to further inform the design of a planned clinical trials. The software was developed alongside hard coded simulations in *R v.3.5.1* ^11^ for trial designs and scenarios that were explored. The trial design simulation code incorporated in the HECT simulator has been validated against multiple trial designs, collectively covering over 1,000 scenarios. The raw code for simulations of the trial designs incorporated in the HECT simulator is available as part of the source code and can be found in the *sim_funs.R* file. Lastly, the software was beta-tested by internal and external colleagues.

## Concluding remarks

We have developed and validated an intuitive highly efficient clinical trial simulator for planning of platform adaptive clinical trials. The software is open-source and can be accessed via any web browser. It therefore caters to clinical trial investigators who do not have the statistical capacity for trial simulations available in their team or who do not have the funds to invest in available commercial software.

## Data availability

### Underlying data

All data underlying the results are available as part of the article and no additional source data are required.

## Software availability

The source code for the software is via GitHub: *https://github.com/MTEKSciencesInc/HECT*

Archived source code at time of publication: http://doi.org/10.5281/zenodo.2552878 ^14^

Licence: GNU General Public License v3.0
