# Supplementary material for: Highly Efficient Clinical Trials Simulator (HECT): Software application for planning and simulating platform adaptive trials
Source: Gates Open Res. 2019 Mar 18;3:780. Originally published 2019 Mar 8. [Version 2] doi: 10.12688/gatesopenres.12912.2 (PMC6556760; doi:10.12688/gatesopenres.12912.2)
Supplement: Peer review history

# Peer review: Reviewer response for version 2

**DOI:** 10.21956/gatesopenres.14050.r27046

**Published:** 2019-06-14

**Authors:** Chris Cameron

**Affiliations:** 1 Cornerstone Research Group Inc., Burlington, ON, Canada

Overall, HECT is an interesting and timely tool. The application of the tool on RShiny will broaden it's applicability and use. Overall, the article is well written, and the sections in the publication are clearly labelled. The tool is intuitive and easy to use. 

 Major comments:

- None. I don't have any major concerns with the indexing of this article.

Minor comments (optional to address):

- The authors may want to consider a more interactive graphics interface such as Highcharter to improve the quality of some of the graphics.

I have read this submission. I believe that I have an appropriate level of expertise to confirm that it is of an acceptable scientific standard.

# Peer review: Reviewer response for version 2

**DOI:** 10.21956/gatesopenres.14050.r27014

**Published:** 2019-06-03

**Authors:** Roger J. Lewis

**Affiliations:** 1 Department of Emergency Medicine, Harbor-University of California, Los Angeles Medical Center, Torrance, CA, USA; 2 Berry Consultants, LLC, Austin, TX, USA; 3 Department of Emergency Medicine, David Geffen School of Medicine, University of California, Los Angeles, Los Angeles, CA, USA

General comments:

- The authors report the capabilities of an open-access, hosted software solution for the simulation of simple platform clinical trials, namely clinical trials that are intended to evaluate a sequence of treatments for a disease, or group of related diseases, when the treatments may not all be available or included initially. This is an area of intense interest and activity within the clinical trials community and, moreover, the lack of readily available software limits the ability of some to evaluate the potential utility of this approach in their research setting. One concern, both in general and specific to this effort, is the challenge of creating software with broad enough capabilities so that the appropriately configured platform trial can be evaluated for each research setting, without creating a system of such complexity that it is inaccessible to all but specialists in this area. Overly simplistic approaches can lead to the erroneous conclusion that a platform approach is not advantageous or, alternatively, has important weaknesses. For example, the performance of response-adaptive randomization (RAR) can be highly dependent on the particular algorithm chosen or on the choice of allocation to the control arm (e.g., it is often important to maintain a fixed or minimum allocation to the control throughout the trial) and thus, without these options, a particular choice of simulation software can lead to erroneous conclusions regarding the advantages and limitation of RAR.
- My most significant concern regarding this effort is the flexibility and capability of the RAR function to capture current “best practices” for RAR, including other allocation rules and fixed or minimum allocation to the ongoing control arm.

Specific comments:

- Abstract: The advantage of the adaptive approach is less to lead to a faster result or approval and, instead, to ensure the trial is of the correct size to get the correct answer. Often this is smaller and faster but, sometimes, it is larger and slower. The goal is to avoid an indeterminate result that leaves the motivating research question unanswered. In my experience, many statisticians have misconceptions regarding the advantages and limitations of adaptive and platform trials. The implication that a lack of familiarity is limited to clinicians is inconsistent with my experience (this should be corrected both in the Abstract and throughout the manuscript). Please note the types of endpoints for which the software is applicable (e.g., continuous and dichotomous, but not time-to-event). What is meant by “validated?”
- Introduction, 2 ^nd^ paragraph: An adaptive platform trial is pre-planned and the adherence to the pre-specified statistical design is critically important to realize the designed operating characteristics. This section should be rewritten to emphasize that pre-specification and adherence to the design are important for all trials; however, in an adaptive platform trial the design includes the pre-specification of changes in trials features, e.g., randomization ratios.
- Introduction, 3 ^rd^ paragraph: As above, simulations are a “black box” to most academic statisticians as well, since simulation is not commonly taught in most PhD statistics programs.
- Introduction, 4 ^th^ paragraph: A concise definition of an adaptive platform design should be provided. Again, the phrase “researchers who are not statisticians” implies that statisticians are generally versed in these methods, which is not the case.
- Implementation, 4 ^th^ paragraph: It would be very helpful to include traditional group-sequential designs (e.g., a multi-arm O’Brien-Fleming with a Lan-DeMets alpha spending function) for comparison, since this is often the type of more traditional option that is being considered.
- Response adaptive allocation, Page 6: Please see comments above regarding the flexibility of the available RAR options.

I have read this submission. I believe that I have an appropriate level of expertise to confirm that it is of an acceptable scientific standard, however I have significant reservations, as outlined above.

## References

[1] SavilleBRBerrySM: Efficiencies of platform clinical trials: A vision of the future. *Clin Trials*.13(3) : 10.1177/1740774515626362 358-66 10.1177/1740774515626362 26908536

[2] WoodcockJLaVangeL: Master Protocols to Study Multiple Therapies, Multiple Diseases, or Both. *New England Journal of Medicine*.2017;377(1) : 10.1056/NEJMra1510062 62-70 10.1056/NEJMra1510062 28679092

# Peer review: Reviewer response for version 2

**DOI:** 10.21956/gatesopenres.14050.r26985

**Published:** 2019-04-12

**Authors:** Howard Thom

**Affiliations:** 1 Bristol Medical School, Population Health Sciences, University of Bristol, Bristol, UK

General comments:

 Thank you very much for the opportunity to review this paper. The authors present a commendable piece of open-source software that allows users to explore statistical properties (incl. type 1 error, power, average sample size, expected costs) of various adaptive trial designs and compare with conventional randomized controlled trial (RCT) designs. This is well motivated by the expense of existing specialist software (e.g. FACTS or ADD-PLAN) and high level of expertise required to perform calculations/simulations without specialist software. The software allows simulation of trials with many interim analyses (determined by sample cumulative sample size) at which adaptive randomization (changes in allocation ratio), platform adaptations (adding a treatment arm), group sequential designs (early stopping for futility, efficacy) can be performed. The analyses are conducted in the Bayesian framework. I have experimented with the software and find it mostly easy to use and fast for the default number of simulations (100). There are a few elements of the software and paper that could be clarified and limitations that should be noted (lack of sample size re-estimation, no use of surrogate outcomes, no correlation between primary and secondary outcomes, too few simulations for Federal Drug Administration (FDA) purposes). I also have a doubt that the tool will be viewed as any less of a black-box than existing software. However, I am confident that this is a useful software and that it is described sufficiently by the paper.

Specific comments:

1. It would be interesting to know how this tool relates to the recent update of the FDA guidelines ( https://www.gmp-compliance.org/gmp-news/revision-fda-guideline-on-adaptive-designs-for-clinical-trials). As they require validated software, perhaps comment on the likelihood that they would accept calculations from this tool?
2. My comment on the FDA may be irrelevant if the primary user base for this tool are researchers in low-and-middle income countries (LMIC), which are the focus of the Gates Foundation. It is here that researchers are likely not to have access to expensive professional services or colleagues with statistical skill necessary to perform simulations/calculations themselves. If this is the primary user base for the tool, it would be good to be explicit in the introduction.
3. Some important adaptive trial designs aren’t included in the tool, such as enriched recruitment designs, use of surrogate outcomes (could be implemented given the inclusion of secondary outcomes), sample size re-estimation. This limitation should be noted in the discussion and it should be stated whether they may be implemented in the future.
4. A general comment from the authors on whether further development of the tool is planned would be interesting.
5. On page 4 of 8, the authors describe a method by which treatment arms are dropped if effects fall below some minimally clinically important effect. I couldn’t find this implemented in the software. If it’s there, it likely needs better signposting.
6. A disadvantage of the simulation is that the primary and secondary outcomes aren’t correlated. Have I missed it somewhere? If it isn’t implemented, please highlight as a limitation.
7. The default number of simulations is 100 but the recent FDA guidelines ( https://www.gmp-compliance.org/gmp-news/revision-fda-guideline-on-adaptive-designs-for-clinical-trials) recommend 100,000, which appears to take an impractical amount of time in the software. There is even a possibility that the internet connection would be interrupted while waiting for completion. This limitation should be noted.
8. The authors claim (particularly in the first sentence of the concluding remarks) that their software is “highly efficient”. Is there some way to justify this claim? Is the simulation design efficient? How does it compare to FACTS or AD-PLAN?
9. For the proportional/dichotomous outcomes, it would be good if the tool allowed users to input odds ratio estimates for non-control treatments, rather than having to provide absolute probabilities for all treatments. This could better match treatment effect estimates from earlier exploratory trials.
10. For response adaptive allocation, only one re-allocation rule (ratio between square roots) is implemented. Are other rules used so rarely that their absence isn’t a limitation? If not, best to highlight the limitation.

I have read this submission. I believe that I have an appropriate level of expertise to confirm that it is of an acceptable scientific standard, however I have significant reservations, as outlined above.

# Peer review: Reviewer response for version 2

**DOI:** 10.21956/gatesopenres.14050.r26986

**Published:** 2019-04-10

**Authors:** Robert A. Beckman, Valeriy Korostyshevskiy

**Affiliations:** 1 Departments of Oncology and of Biostatistics, Bioinformatics, and Biomathematics, Lombardi Comprehensive Cancer Center & Innovation Center for Biomedical Informatics (ICBI), Georgetown University Medical Center (GUMC), Washington, DC, USA; 2 Department of Biostatistics, Bioinformatics, and Biomathematics, Lombardi Comprehensive Cancer Center, Georgetown University Medical Center (GUMC), Washington, DC, USA

Thorlund *et al. *have developed a simulator of adaptive and platform trials (collectively known as highly efficient clinical trials, HECT) which runs as a web application and has a very accessible, sensibly laid out graphical user interface. HECT are underutilized in part because of the need to perform complex simulations to assess their performance characteristics. These simulations often exceed the statistical resources and/or expertise of people or organizations that might otherwise use HECT to improve their cost efficiency of performing clinical trials.

 The article meets all criteria for approval in our judgment.

**Valeriy Korostyshevskiy:** The article is very cleanly written; it is easy to follow the logic of the design and understand the principles of how the software operates. I have noticed some typos:

- Page 4, 1st paragraph: "Figure 2a display **s**..."
- Page 4, 3rd paragraph: "...if the probability that it is superior to all other treatments fall **s** below..."
- Page 6, second column, 3rd paragraph: "...the input field will automatically change to match the select **ion**."

Thoughts on the tool itself:

1. I played around with the tool and found it very user friendly, intuitive, and easy to use. 
2. It would probably still require some learning period for someone using such a tool for the first time or not having the background, but I do not envision such a learning period to be long (see comment 1).
3. The tool is self-contained - the manual and the explanations of how things work add value in this regard. This is a very important feature. 
4. This software runs on R on a remote server, so the simulation time could be impacted by these two factors. (R is not the fastest although some of the routines maybe hard-coded using standard C or Fortran routines - these details are not presented by the authors (nor do I think they should be presented in this article)). A researcher could eliminate the potential remote server issue by downloading the package and using it locally, however, given current internet speeds, this is unlikely to result in substantial computation time decrease. 
5. The availability of the package for downloading and using locally is a big plus.
6. GUI is clean, right to the point, without unnecessary bells and whistles. One suggestion to the authors is to modify the HTML code so that the browser tab header would reflect the tool's name. Currently it has the HTML line with the name of the KI image used on the top of the page ().

**Robert A. Beckman:** I think the article is excellent. 

1. Type I and type II errors can be defined in various ways for a multi-arm study. Are they defined in this simulation by arm or total? What is the null hypothesis?
2. It would be useful to see a comparison of power/cost ratio (efficiency) under the constraint of a comparable type I error. This would easily quantify the benefit of HECT. The example shows a higher type I error for HECT. Is this just a fluctuation that would go down for a sufficiently large number of trials? 
3. Typo in the section on "response adaptive allocation", referring to it initially as "response adaptive adaptation."

We have read this submission. We believe that we have an appropriate level of expertise to confirm that it is of an acceptable scientific standard.
